# MIRROR-TCM TRIAL: THE INFLUENCE OF PATIENT-LEVEL FACTORS ON ACUTE CARE RESOURCE USE OF OLDER ADULTS

**DOI:** 10.1093/geroni/igad104.3473

**Published:** 2023-12-21

**Authors:** Onome Osokpo, Karen Hirschman, Monica Ahrens, Wenyan Ji, Alexandra Hanlon, Mary Naylor

**Affiliations:** University of Pennsylvania, Philadelphia, Pennsylvania, United States; University of Pennsylvania, Philadelphia, Pennsylvania, United States; Virginia Tech, Blacksburg, Virginia, United States; Virginia Tech, Blacksburg, Virginia, United States; Virginia Tech, Blacksburg, Virginia, United States; University of Pennsylvania, Philadelphia, Pennsylvania, United States

## Abstract

Adverse Social Determinants of Health (SDoH) impact post-hospitalization outcomes (e.g., frequent acute care resource use: emergency department [ED] visits, rehospitalizations). The Transitional Care Model Intervention (TCM), an advanced practice registered nurse–led team-based care-management strategy, has demonstrated improved outcomes among older adults throughout transitions from hospital to home. Yet, little is known about the relationship between specific patient-level factors (i.e., demographic, clinical, SDoH) and acute care resource use (i.e., counts of rehospitalizations and ED visits) among hospitalized older adults with multiple chronic conditions who receive the TCM intervention. A total of 480 patients were randomized to receive the TCM in the MIRROR-TCM trial conducted from September 2020 to March 2023 at three diverse health systems in four states. Using multivariable generalized linear mixed effects regression with backward elimination, we examined the relationship between demographic (e.g., biological sex), clinical (e.g., counts of chronic conditions, etc.), SDoH characteristics (e.g., transportation, etc.), and acute care resource use during the intervention. Compared to those without housing concerns, older adults with housing concerns had 2.4 times higher rates of ED visits (p=0.049) and 2.2 times higher rates of rehospitalizations (p=0.005). Those who lacked transportation for medical appointments had 2.5 times higher ED visit rates (p=0.021) and 1.5 times higher rehospitalization rates (p=0.041). For each additional comorbidity, the rate of rehospitalization increased by a multiple of 1.08 (p=0.008). Understanding the adverse SDoH factors and increasing clinical complexity impacting resource use and tailoring evidence-based transitional care interventions to mitigate these factors to improve posthospitalization outcomes is critical.

